# A High-Throughput, High-Content Analysis of Dopaminergic Neurodegeneration in *Caenorhabditis elegans* Exposed to Per- and Polyfluoroalkyl Substances

**DOI:** 10.3390/toxics14040278

**Published:** 2026-03-26

**Authors:** David Benson, Seth Currie, Jia-Sheng Wang, Lili Tang

**Affiliations:** 1 Interdisciplinary Toxicology Program, University of Georgia, Athens, GA 30602, USA; david.benson@uga.edu (D.B.); sdc80106@uga.edu (S.C.); jswang@uga.edu (J.-S.W.); 2 Department of Environmental Health Science, College of Public Health, University of Georgia, Athens, GA 30602, USA

**Keywords:** *Caenorhabditis elegans*, high-throughput, high-content imaging, PFAS, dopaminergic neurotoxicity, dopamine-dependent behavior, environmental pollutants

## Abstract

Dopaminergic neurodegeneration is a hallmark of Parkinson’s disease (PD), and environmental contaminants have been implicated in disrupting dopaminergic pathways. However, practical in vivo workflows for rapid, standardized, and accessible assessment of dopaminergic neurotoxicity remain limited. In this study, we built on our laboratory’s established high-throughput framework and implemented a high-content imaging workflow to quantify DA neurodegeneration in *Caenorhabditis elegans* following exposure to representative per- and polyfluoroalkyl substances (PFAS). We evaluated the neurotoxic effects of perfluorooctanesulfonic acid (PFOS), perfluorooctanoic acid (PFOA), perfluorohexanesulfonic acid (PFHxS), perfluorohexanoic acid (PFHxA), and three PFAS mixtures with environmentally relevant component ratios. Functional relevance was assessed using dopamine-dependent behavioral endpoints, including basal slowing response (BSR) and area-restricted search (ARS). PFOS exhibited the greatest potency, followed by PFHxS, PFHxA, and PFOA, based on morphological degeneration and benchmark concentration modeling. Structural neuronal damage was significantly associated with behavioral impairment. Under mixture conditions, neurotoxicity was more strongly associated with PFOS molar fraction than with total PFAS concentration (ΣPFAS), suggesting a composition-dependent toxicity profile. Collectively, these findings establish a scalable in vivo framework for assessing PFAS-induced dopaminergic neurotoxicity and support the potential use of this platform for screening environmental pollutants with dopaminergic neurotoxic potential.

## 1. Introduction

Parkinson’s disease (PD) is a progressive neurodegenerative disorder characterized by the selective loss of dopaminergic (DA) neurons in the substantia nigra and the formation of Lewy bodies containing aggregated α-synuclein [1]. Behind Alzheimer’s disease, PD is the second most common neurodegenerative disorder in the United States, projected to affect 1.2 million Americans by 2030, with an estimated 90,000 new cases diagnosed each year [2]. The most common symptoms of PD such as gait impairments, tremors, rigidity, akinesia and bradykinesia stem from the progressive degeneration of DA neurons in the brain [3,4]. While genetic predisposition accounts for a subset of PD cases, mounting evidence implicates environmental factors as major contributors to its etiology [2]. Epidemiological and experimental studies have demonstrated strong associations between PD incidence and exposure to environmental toxicants such as pesticides (e.g., rotenone, paraquat), industrial solvents like trichloroethylene (TCE), and common air pollutants [5]. For example, environmentally relevant polystyrene nanoplastic exposure was found to induce Parkinson’s-like neurotoxicity in *C. elegans* via the suppression of DA neuron fluorescence (CEP and PDE) and the elevation of α-synuclein aggregation. This suppression of DA transmission gives rise to the loss of the dopamine-founded behavior basal slowing response [6]. Similarly, silica nanoplastic exposure from heavy traffic-generated air was found to elevate the dendritic beading (common neurodegenerative phenotype) of DA neurons while lowering thrashing rates in the same strain [7]. These findings suggest that environmental exposures may contribute to DA neurodegeneration through mechanisms such as oxidative stress, mitochondrial dysfunction, and neuroinflammation. However, despite growing awareness of environmentally linked neurotoxicity, the role of many emerging contaminants—particularly per- and polyfluoroalkyl substances (PFAS)—in DA neurodegeneration remains largely unexplored.

PFAS, also referred to as “forever chemicals”, are a widely distributed and persistent class of >12,000 man-made chemicals used is the mass-manufacturing of oil, fire, water and stain-resistant products [8,9]. Their carbon–fluorine backbones (–CnF_2n+1_–) confer exceptional stability, leading to environmental persistence and bioaccumulation [10,11,12,13]. PFAS are commonly used in the production of non-stick cookware, furniture, food packaging, and aqueous film-forming foams (AFFFs). Repeated AFFF use at military bases and airports has led to widespread contamination of soil and water, resulting in elevated PFAS levels in drinking water and the food chain [14,15]. Consumption of contaminated food/water supply in addition to the use of any PFAS-infused cookware or food packaging can result in serum PFAS levels well above advisory limits. Accumulating evidence suggests that PFAS can cross the blood–brain barrier and accumulate in the brain [16,17,18], which can lead to neurotoxic outcomes including impaired spatial learning/memory behaviors [10,19], motor development [20] and neurodevelopment [10,21,22,23,24]. PFAS exposure has been linked to perturbations in cholinergic, GABAergic, and glutamatergic neurotransmission [25,26,27]. Yet its effects on dopaminergic signaling—a key target in PD—remain poorly defined. Understanding whether PFAS contributes to DA neuron degeneration and PD-like pathology is therefore critical.

The nematode *Caenorhabditis elegans* (*C. elegans*) is an established model organism in neurotoxicology due to its simple yet highly conserved nervous system. This 1 mm transparent worm possesses 302 neurons with a fully mapped connectome, enabling direct visualization of neuronal structure and function [28]. Its short lifespan, high fecundity, and ease of maintenance make it a cost-effective and ethically favorable alternative to mammalian models for neurotoxicity assessment [29]. Its transparency allows high-resolution in vivo imaging, while its genetic tractability and conserved neurotransmission pathways permit mechanistic studies relevant to human health. The BZ555 strain is a transgenic *C. elegans* line widely used for studying DA neurotoxicity because it enables direct visualization of DA neurons in vivo. This strain carries the *egIs1* [*dat-1p::GFP*] transgene, which drives GFP expression specifically in the worm’s dopaminergic neurons. Owing to the transparency of *C. elegans*, BZ555 allows fluorescent imaging and quantitative assessment of neuronal integrity in living animals and has been widely used to evaluate dopaminergic damage induced by neurotoxicants and environmental contaminants, including PFAS [30,31,32]. Neurodegeneration in this strain is typically characterized by morphological defects, such as loss of neuronal cell bodies and neurite blebbing, and can also be linked to dopamine-dependent behavioral deficits. Despite its advantages, current *C. elegans* neurotoxicity studies often rely on low-throughput manual imaging and scoring, which are time-consuming, subjective, and prone to bias. High-throughput, high-content (HTHC) imaging platforms overcome these limitations by combining automated sample handling, immobilization, and quantitative fluorescence imaging analysis. Such systems greatly enhance scalability, precision, and reproducibility in neurotoxicity assessment [33,34].

In this study, we built on our laboratory’s established high-throughput framework and implemented a HTHC platform integrating COPAS BioSort worm dispensing, simultaneous microfluidic immobilization of up to 40 worms using vivoChip-2x, and quantitative imaging of dopaminergic neurons in transgenic *C. elegans* (BZ555) using Cytation 5. A key advantage of this workflow is that it enables rapid, high-throughput, and standardized neuronal imaging without reliance on confocal fluorescence microscopy, which is often costly and limited to specialized imaging facilities. Four representative PFAS (PFOS, PFOA, PFHxS, and PFHxA) were selected based on their prevalence in AFFF-impacted environments. To complement structural assessment, dopamine-dependent behavioral endpoints were evaluated to determine functional consequences of exposure. This integrated approach enables practical assessment of PFAS-induced DA neurotoxicity and supports broader application of the platform for environmental neurotoxicity screening.

## 2. Materials and Methods

### 2.1. Chemicals

**Figure 1:**
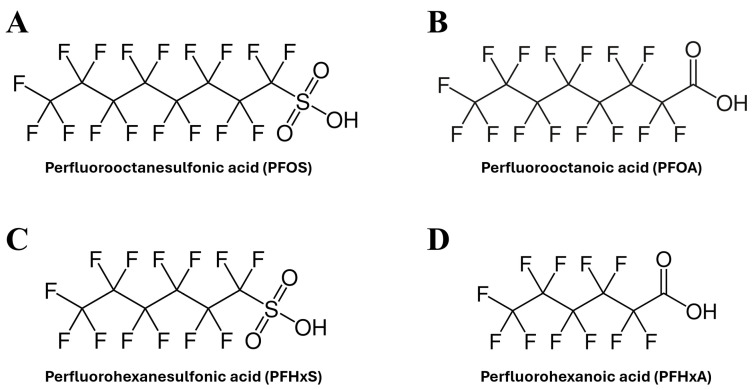
The molecular structure of the four chosen PFAS: PFOS (**A**), PFOA (**B**), PFHxS (**C**), PFHxA (**D**).

Four PFAS were selected for this study, representing major PFAS commonly detected in AFFF-contaminated water supplies [35,36]: perfluorooctanesulfonic acid (PFOS), perfluorooctanoic acid (PFOA), perfluorohexanesulfonic acid (PFHxS), and perfluorohexanoic acid (PFHxA). The molecular structures of these four PFAS are shown in Figure 1.

Analytical-grade PFOA (95%, CAS No. 335-67-1) was obtained from Sigma Aldrich (St. Louis, MO, USA), PFOS (98%, CAS No. 1763-23-1) and PFHxS (95%, CAS No. 355-46-4) were purchased from Synquest Laboratories, Inc. (Alachua, FL, USA), and PFHxA (98%, CAS No. 307-24-4) was purchased from Astatech Inc. (Bristol, PA, USA). Stock solutions at 1 M were prepared in dimethyl sulfoxide (DMSO), and working solutions were diluted using k-medium (32 mM KCl and 51 mM NaCl) [37] containing *Escherichia coli* OP50 (1 mg/mL) as a food source [38].

### 2.2. C. elegans Maintenance and Exposure

The transgenic *C. elegans* strain, BZ555 [Pdat-1::GFP], and the *Escherichia coli* strain OP50 were obtained from the Caenorhabditis Genetics Center (Minneapolis, MN, USA). This strain is often used in studies investigating the neurotoxic effects of environmental pollutants, particularly via the phenotypic alterations of DAc neurons [7,39,40].

The nematodes were maintained on nematode growth medium (NGM) agar plates seeded with OP50 at 25 °C and were transferred to fresh plates every 3–4 days [41,42]. Synchronized populations were prepared by isolating eggs with sodium hypochlorite treatment, followed by hatching in K-medium overnight with shaking to obtain L1 growth-arrested larvae [43,44]. L4-stage worms were produced by allowing synchronized L1 worms to grow on NGM for 28–30 h. For toxicity testing, age-synchronized worms were washed off the NGM plates using K-medium, concentrated by centrifugation at 2500× *g* for 10 min, and subsequently exposed to different concentrations of individual PFAS or PFAS mixtures. The working solutions were diluted from stock solutions at six concentrations (0~500 μM), which were designed and refined based on the results from previous studies [10,41].

### 2.3. PFAS Selection and Mixture Construction

As the persistence of PFAS seeped into soil and water systems from regular aqueous film-forming foams (AFFF) discharges, significant concerns were raised about the serious health burden on military bases and nearby communities. In this study, four representative PFAS, PFOS, PFOA, PFHxS and PFHxA, were selected based on their high detection frequency and concentrations in surface waters near AFFF-impacted areas [35,45], as well as their elevated serum levels reported among U.S. Air Force servicemen [46]. Because a primary objective of this study is to establish and validate an efficient HTHC imaging platform for assessing DA neurodegeneration in *C. elegans*, relatively high concentrations were used in the single-PFAS exposures (100–500 µM) to induce measurable neurodegeneration and evaluate the performance of the imaging platform and subsequent grading system.

Because AFFF formulations are complex mixtures of fluorinated surfactants, assessing the toxicity and risk of PFAS mixtures under realistic exposure scenarios is essential. Previous studies have shown that simplified laboratory mixtures can effectively mimic the chemical composition of AFFF-contaminated environments [35,45,47]. Therefore, three PFAS mixtures (M1–M3; Table 1) were designed to represent stepwise levels of chemical complexity while maintaining environmentally relevant component ratios. Although the absolute molar concentrations of individual PFAS in these mixtures were relatively high, for the reasons described above, the proportional composition of each mixture was based on ratios reported in AFFF-impacted water sources [45]. Mixture 1 (M1) consisted of PFOS + PFOA in a normalized molar ratio of 0.88:0.12, representing the dominant sulfonate and carboxylate constituents typically present in AFFF residues. Mixture 2 (M2) included PFOS + PFHxS + PFOA in a ratio of 0.60:0.30:0.10, reflecting the composition frequently detected in human serum and groundwater from AFFF-impacted locations. Mixture 3 (M3) comprised PFOS + PFHxS + PFHxA + PFOA in a ratio of 0.56:0.28:0.09:0.07, closely approximating the median environmental distribution of these PFAS across contaminated sites. Each mixture was prepared from individual PFAS stock solutions and diluted with exposure medium to achieve final total PFAS concentrations (ΣPFAS) of 100 µM and 200 µM. Vehicle controls contained the same DMSO concentration (<0.05% *v*/*v*).

### 2.4. Developing the HTHC Imaging Platform

**Figure 2:**
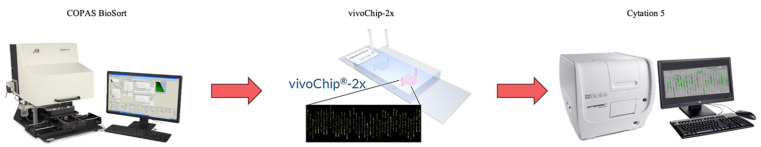
The proposed HTHC imaging platform.

High-throughput, high-content (HTHC) imaging of DA neurons in *C. elegans* was performed using an integrated workflow consisting of COPAS BioSort (Union Biometrica, Holliston, MA, USA) worm dispensing, vivoChip-2x microfluidic immobilization of up to 40 worms, and Cytation 5 fluorescence imaging (Figure 2). Although these individual components are commercially available, their implementation within a single workflow enabled rapid, standardized, and accessible neuronal assessment compared with traditional low-throughput, one-by-one observation. A key feature of this platform is that it allows efficient neuronal imaging without reliance on confocal fluorescence microscopy, thereby improving practicality for routine laboratory use.

**Figure 3:**
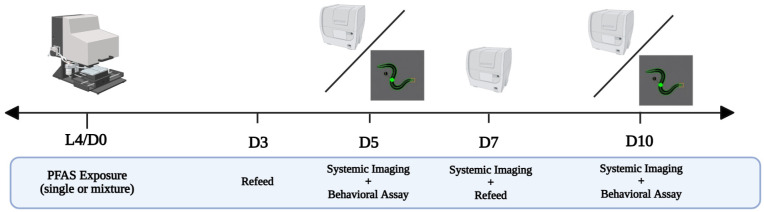
Exposure and assay timeline of both the HTHC imaging platform and behavioral analysis.

For exposure experiments, approximately 120 L4 worms were transferred into a 96-well plate at a density of 30 worms per well and exposed to PFAS-containing food in liquid medium. To maintain food availability throughout the exposure period, 100 μL of food was added to each well on days 3 and 7 post-exposure. To prevent progeny production, 90 μM floxuridine (FUdR) was added to each well on the day of exposure. Worms were imaged on days 5, 7, and 10 post-exposure. Behavioral assays were conducted using the same exposure conditions and experimental timeline (Figure 3).

At each imaging time point, worms from four replicate wells were pooled into a single Eppendorf tube per concentration and briefly centrifuged to obtain a pellet. Using a 10 mL syringe and the transfer tubing from the vivoChip-2x kit (vivoVerse, Austin, TX, USA), approximately 40 worms were loaded into the chip’s immobilization wells. Immobilization was achieved through pressure activation of the connected vivoCube+ chamber, ensuring stable positioning for high-resolution imaging. The vivoChip-2x was then mounted into the Cytation 5 imaging system (Agilent, Santa Clara, CA, USA) using a glass-slide adapter (Agilent, Santa Clara, CA, USA), and GFP fluorescence images of the six head-region DA neurons were acquired at 60× magnification. Approximately 25 worms per treatment group were analyzed in at least three independent experiments.

**Figure 4:**
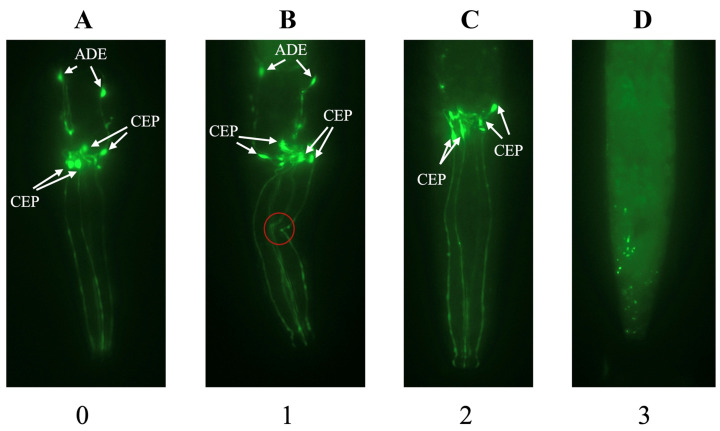
Representative images of the scoring tiers with annotations. An image scored a “0” due to 6 visible intact neurons without morphological blemishes (**A**). An image scored a “1” due to 6 visible intact neurons with a morphological blemish (kink, red circle) (**B**). An image scored a “2” due to 4 visible intact neurons (**C**). An image scored a “3” due to 0 visible intact neurons (**D**).

Neuronal damage was evaluated using a revised 0–3 scoring system (Table 2). Scores were assigned on a whole-animal basis rather than to individual neurons. The term “intact neuron” denotes a clearly visible soma connected to its corresponding axon. Kinks and blebs are included under the score “1” criteria as morphological indicators of early neurodegeneration, distinguishing healthy neurons from those exhibiting structural damage [33,48,49]. Representative images for each score category are shown in Figure 4. To improve scoring consistency, raters were trained in advance using representative images and standardized scoring criteria.

### 2.5. DA-Dependent Neuronal Behavioral Analysis

For verification of the morphological assessment, two dopamine-dependent foraging behaviors, area-restricted searching (ARS) and basal slowing response (BSR), were assessed to evaluate functional impairments of dopaminergic neurons. ARS represents food-searching behavior in the absence of resources, characterized by reduced turning frequency and straighter movement paths, whereas BSR reflects decreased locomotion in the presence of food [50,51,52,53,54]. Disruption of dopaminergic signaling is expected to reduce absolute peristaltic speed (µm/s) and turn count. Single-compound PFAS assays were performed on days 5 and 10 post-exposure, and mixture assays on day 10. On each test day, worms from six replicate wells were pooled and centrifuged to obtain a pellet, which was evenly dispensed around OP50-seeded nematode growth medium (NGM) plates. After drying and resuming crawling, worms were transferred to mini OP50-seeded and non-seeded NGM plates and acclimated for 1 h. Behavioral recordings were acquired for 45 s using the WormLab system (2023, MBF Bioscience, Williston, VT, USA). The software automatically quantified absolute peristaltic speed and turn count.

### 2.6. Benchmark Dose Calculation

The benchmark concentration at 10% (BMC10) and its lower confidence limit (BMCL) were determined using PROASTweb software (version 70.1; https://proastweb.rivm.nl/, accessed on 8 April 2025). The data from each assay were summarized in Excel, including PFAS concentration levels and their corresponding response effects, then imported into PROASTweb for quantitative modeling. Model fitting was performed using the maximum likelihood estimation (MLE) method. Benchmark concentration calculations were conducted under default settings with a 95% confidence level, assuming constant variance. Model selection was based on the lowest Akaike Information Criterion (AIC) value (*p* < 0.05). Final BMC_10_ and BMCL estimates, along with model parameters and fit statistics, were reported for each sample.

### 2.7. Statistical Analysis

All graphs were made using GraphPad Prism (ver.10.2.0). The HTHC imaging data were analyzed using ordinal logistic regression using R Studio (packages: “ordinal”, “rcompanion”, “MASS”, “brant”; ver.2024.12.0+467). To correct for type I errors, considering the multiple comparisons within the ordinal logistic regression, significance was corrected using the Holm-Bonferroni method. Behavioral data were analyzed using 2-way analysis of variance (ANOVA) with a significance level of α = 0.05 in GraphPad Prism. Spearman’s rank correlation coefficients were calculated in RStudio (version 2025.09.1+401 using packages dplyr and broom).

## 3. Results

### 3.1. HTHC Imaging of Individual PFAS Exposure

To evaluate the proposed HTHC imaging approach, we examined phenotypic DA neuron damage in *C. elegans* following exposure to PFOS, PFOA, PFHxS, and PFHxA, across six concentrations (0–500 µM). Across all control groups, most worms retained intact DA neurons, although a small proportion exhibited score 1 and occasionally score 2 phenotypes. In our scoring system, score 1 reflects relatively mild morphological alterations rather than substantial neuronal damage or overt toxicity. In addition, because animals were evaluated on day 10, some age-related DA neurodegenerative changes may also have contributed to these low-level background findings in control worms.

**Figure 5:**
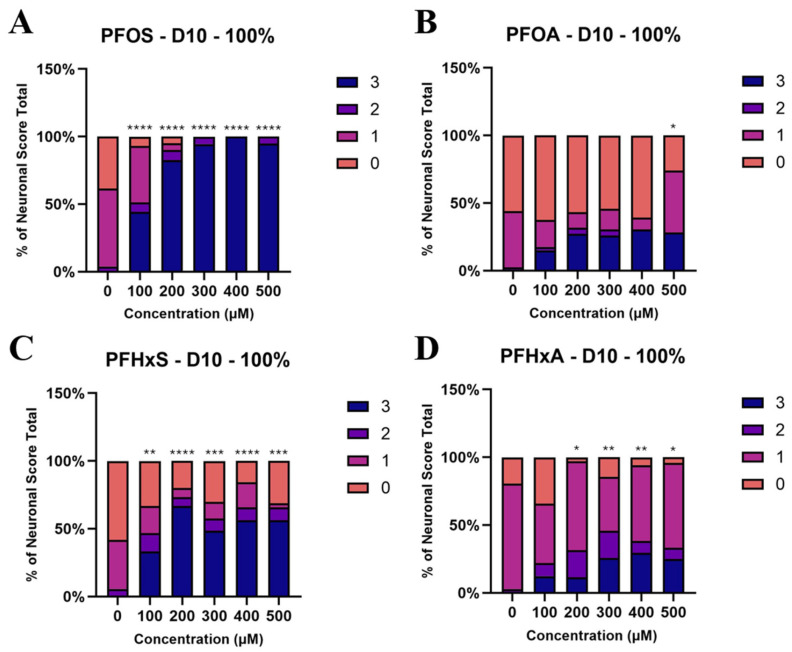
Ordinal logistic regression comparing the frequencies of the imaging scores of the exposure groups (100–500 µM) to the control (0 µM). Neuronal score distribution for PFOS (**A**), PFOA (**B**), PFHxS (**C**) and PFHxA exposure (**D**). * *p* < 0.5, ** *p* < 0.01, *** *p* < 0.001, **** *p* < 0.0001.

At day 10 of treatment, PFOS (Figure 5A) caused pronounced neurodegeneration even at 100 µM (*p* < 0.0001), with the proportion of severely damaged neurons (score 3) increasing from 0% to 44.19% and intact neurons (score 0) disappearing at ≥300 µM. In addition, worms exposed to PFOS at ≥300 µM did not retain more than four intact DA neurons, consistent with the complete absence of score 0 and score 1 animals in these treatment groups. Given its established DA neurotoxicity [55], PFOS also served as a useful reference compound in this study.

In contrast, PFOA (Figure 5B) produced minimal damage at 100–400 µM but moderate degeneration at 500 µM (*p* < 0.05). Specifically, score 0 frequencies dropped from 56.10% (0 µM) to 26.09% in 500 µM PFOA-exposed worms. At this highest concentration, the frequency of score 0 animals decreased from 56.10% in controls to 26.09%, while the frequency of score 3 animals increased by 28.26% relative to controls. PFHxS (Figure 5C) induced moderate neurodegeneration at 100 µM (*p* < 0.01) and severe effects at concentrations ≥200 µM. At 400 µM PFHxS, the proportion of score 0 animals decreased to 15.63%, compared with 58.33% in the control group, indicating substantial loss of neuronal integrity. PFHxA (Figure 5D) exposure caused moderate to severe damage above 100 µM, with score 0 frequencies dropping from 19.44% to 6.80% and score 3 rising to 25% at 500 µM (*p* < 0.01). According to the benchmark dose (Table 3), the neurotoxicity rank was PFOS > PFHxS > PFHxA > PFOA, consistent with previous reports [56,57].

**Figure 6:**
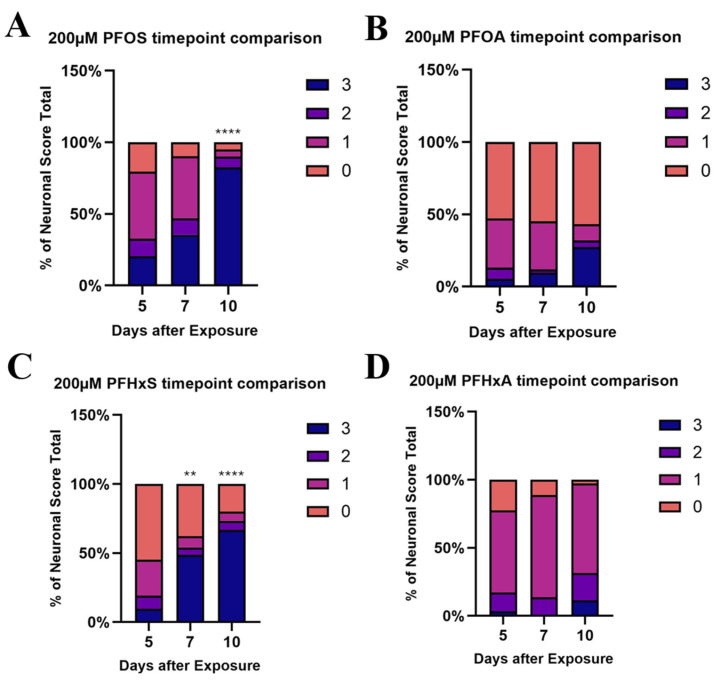
Ordinal logistic regression comparing the frequencies of the imaging scores of the 7th and 10th days following exposure to the 5th. Timepoint comparisons for PFOS (**A**), PFOA (**B**), PFHxS (**C**) and PFHxA exposure (**D**). ** *p* < 0.01, **** *p* < 0.0001.

Time-course analysis (Figure 6) at 200 µM revealed significant increases in neuronal damage on day 10 versus day 5 for PFOS, PFHxS, and PFHxA, but not for PFOA. PFHxS showed the strongest time-dose dependence, while PFOS displayed rapid onset of damage. These findings underscore the importance of in vivo temporal evaluation for understanding PFAS-induced dopaminergic neurotoxicity.

### 3.2. Behavioral Validation of Dopaminergic Imaging Findings

To validate the imaging-based neurotoxicity trends, two dopamine-dependent foraging behaviors—basal slowing response (BSR) and area-restricted searching (ARS)—were assessed using absolute peristaltic speed (PS) and turn count (TC) measurements, respectively. Considering PFAS are not known to alter *E. coli* viability in laboratory conditions or in the gut microbiome of PFAS-exposed organisms [58,59], these endpoints serve as functional and representative readouts of DA neuron integrity in *C. elegans*. Because of the severe toxicity previously observed with PFOS, exposure concentrations were reduced to 0–100 µM for this compound.

**Figure 7:**
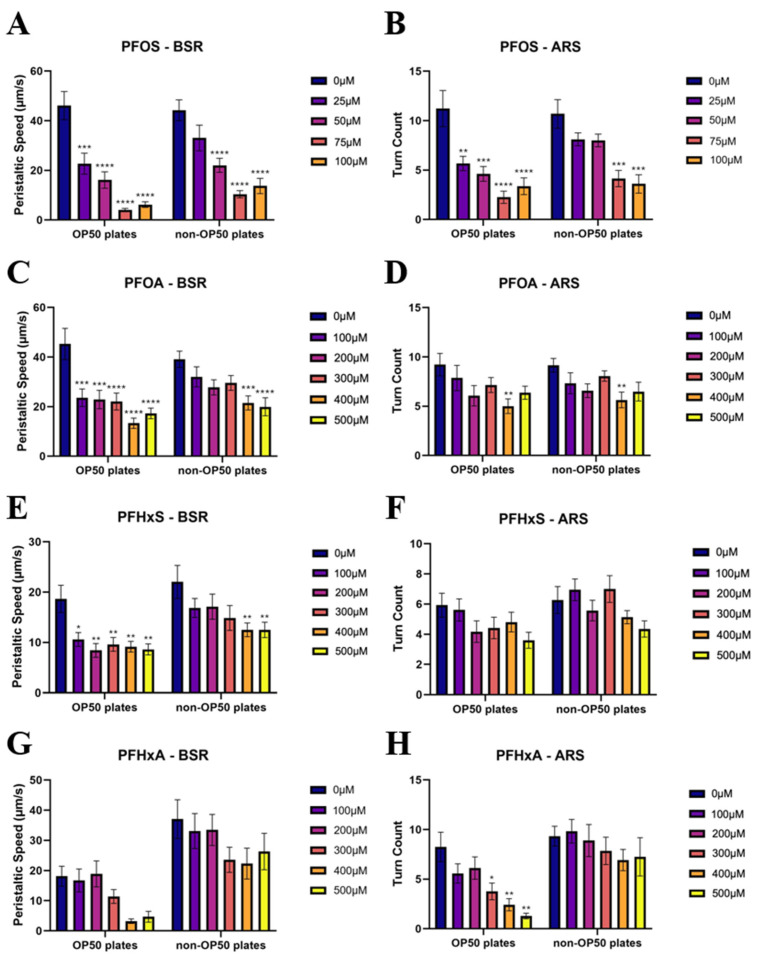
Two-way ANOVA analysis comparing the day ten endpoints of the single-PFAS exposure groups to the control of the respective plate type. PFOS exposure to absolute peristaltic speed (**A**) and turn count (**B**). PFOA exposure to absolute peristaltic speed (**C**) and turn count (**D**). PFHxS exposure to absolute peristaltic speed (**E**) and turn count (**F**). PFHxA exposure to absolute peristaltic speed (**G**) and turn count (**H**). * *p* < 0.5, ** *p* < 0.01, *** *p* < 0.001, **** *p* < 0.0001.

Figure 7 summarizes the behavioral responses to each PFAS. PFOS (Figure 7A,B) caused pronounced behavioral impairment with PS significantly reduced at ≥50 µM (*p* < 0.0001) and TC declines detected at 25 µM (OP50) and 75 µM (non-OP50). PFOA (Figure 7C,D) reduced PS at ≥100 µM on OP50-seeded plates and ≥400 µM on non-OP50 plates, with minimal TC effects. PFHxS (Figure 7E,F) induced PS reductions at all concentrations on OP50-seeded plates and ≥300 µM on non-OP50 plates (100 µM, *p* < 0.05; 200–500 µM, *p* < 0.01) without significant TC changes. PFHxA (Figure 7G,H) reduced PS at all doses and TC at 300–500 µM (300 µM, *p* < 0.05; 400–500 µM, *p* < 0.01). In summary, PFOS caused the most pronounced and concentration-dependent behavioral deficits, whereas PFHxS and PFHxA induced moderate impairments, and PFOA exhibited only limited effects at higher exposure levels.

Spearman’s rank correlation analysis demonstrated insignificant positive associations between DA neuron damage and locomotor behavioral impairment across the four single-PFAS exposures. On day 10, strong insignificant correlations between BSR and DA neuron loss were observed for PFOS (ρ = 0.9, *p* = 0.0833, *n* = 6), PFHxS (ρ = 0.9, *p* = 0.0833, *n* = 6) and PFHxA (ρ = 0.8, *p* = 0.133, *n* = 6). On day 10, a significant positive correlation between ARS and DA neuron loss was observed for PFOS (ρ = 1, *p* = 0.0167, *n* = 6) while moderate to strong insignificant correlations among the two variables were observed for PFHxS (ρ = 0.5, *p* = 0.45, *n* = 6) and PFHxA (ρ = 0.9, *p* = 0.0833, *n* = 6). While the low sample count can give rise to the lower *p* values, the overall coupling between structural damage and functional impairment underscores the reliability of the HTHC platform for assessing DA neurodegeneration in *C. elegans*.

### 3.3. Neurological Disorder of Constructed PFAS Mixtures

#### 3.3.1. Neuronal Integrity Following PFAS Mixture Exposure

**Figure 8:**
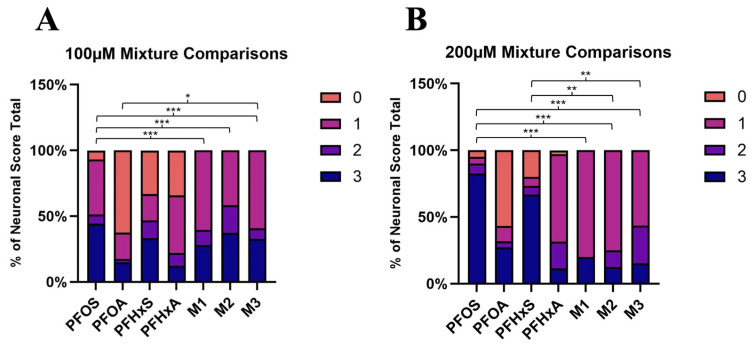
Ordinal logistic regression comparing the frequencies of the mixture imaging scores to the single-PFAS exposures of the same concentration. Mixture comparisons for 100 µM mixtures (**A**) and 200 µM mixtures (**B**). * *p* < 0.5, ** *p* < 0.01, *** *p* < 0.001.

To evaluate the neurotoxic potential of PFAS mixtures, DA neuron integrity was quantified at Day 10 following exposure to three PFAS combinations (M1, M2, and M3) using the established HTHC platform. Neuronal morphology was scored on a four-point scale (0–3), with results expressed as the percentage of the total neuronal score distribution (Figure 8). At a total concentration of 100 µM (ΣPFAS), all three mixtures showed reduced neurodegeneration compared with the 100 µM PFOS-only group. This pattern was reflected by a marked decrease in the frequency of score 3 animals (severe degeneration), from 44.2% in the PFOS-only group to 16.7% in M1, 19.3% in M2, and 20.3% in M3. A similar and more pronounced pattern was observed at 200 µM ΣPFAS. In the PFOS-only group, the frequency of score 3 animals reached 82.5%, whereas the corresponding mixture groups showed substantially lower frequencies: 12.5% for M1, 11.1% for M2, and 9.1% for M3. Additional statistical analysis showed no significant differences between 100 and 200 µM within the same mixture group (M1, *p* = 0.885; M2, *p* = 0.333; M3, *p* = 0.964).

Because the PFOS concentration was lower in the mixture treatments than in the PFOS-only treatment, and neurodegeneration was correspondingly reduced under mixture exposure, these findings suggest that the neurotoxic effects of the mixtures may be strongly influenced by the PFOS component. Collectively, these data indicate that, in mixtures formulated with environmentally relevant component ratios, PFOS composition may be an important determinant of neurotoxic outcomes.

#### 3.3.2. Behavioral Validation of Neuronal Integrity Following PFAS Mixture Exposure

**Figure 9:**
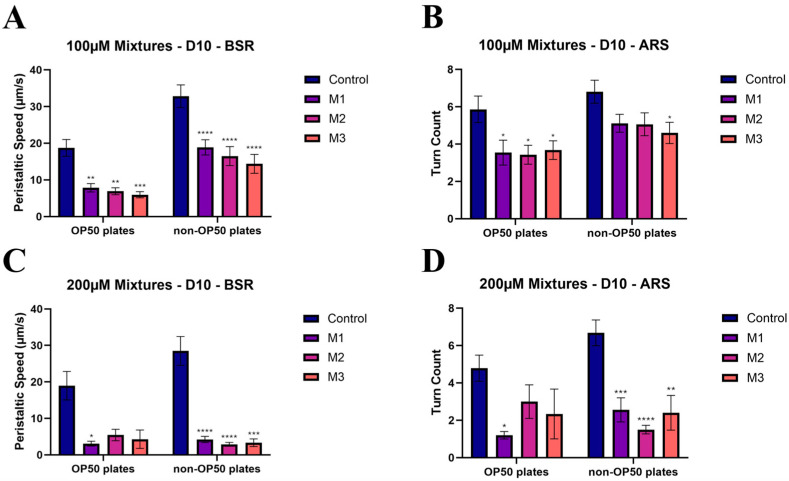
2-way ANOVA analysis comparing the day ten endpoints of the mixture exposure groups to the control of the respective plate type. Set 1 mixture exposures to absolute peristaltic speed (**A**) and turn count (**B**). Set 2 mixture exposures to absolute peristaltic speed (**C**) and turn count (**D**). * *p* < 0.5, ** *p* < 0.01, *** *p* < 0.001, **** *p* < 0.0001.

To evaluate the functional consequences of mixture-induced neurotoxicity, peristaltic speed (PS) and turn count (TC) were monitored in nematodes exposed to environmentally relevant PFAS mixtures (ratios detailed in Table 2). Figure 9 summarizes the locomotor (PS) and exploratory (TC) behavioral profiles for these mixture cohorts.

A consistent, cumulative reduction in PS was observed among worms exposed to the (ΣPFAS) mixtures on both OP50-seeded and non-seeded plates (Figure 9A). Similarly, TC values exhibited a comparable decrease across all mixture treatments on OP50-seeded plates (Figure 9B). Notably, behavioral performance remained relatively uniform across the different mixture compositions (M1–M3) within each set. This consistency, despite the varying ratios of PFOA, PFHxS, and PFHxA, further suggests that PFOS may play a dominant role in the neurotoxic profile of mixtures formulated with environmentally relevant component ratios and may contribute most substantially to the observed functional impairment. These behavioral trends align with the limited but detectable neuronal damage revealed by the HTHC imaging assay.

## 4. Discussion

In this study, we built on our laboratory’s established high-throughput framework and implemented a HTHC imaging workflow for quantitative assessment of DA neurodegeneration in *C. elegans* following exposure to representative PFAS. By integrating automated worm dispensing, microfluidic immobilization, high-resolution GFP imaging, and dopamine-dependent behavioral assays, this platform provides a scalable in vivo approach for neurotoxicity screening while preserving structural–functional coupling [33,34,60]. The strong correlations observed between neuronal damage scores and behavioral impairments support the biological relevance of the imaging endpoints and demonstrate that this system captures functionally meaningful DA dysfunction [49].

Among the four PFAS tested, a consistent potency ranking was observed: PFOS > PFHxS > PFHxA > PFOA. PFOS produced pronounced morphological degeneration and behavioral impairment at comparatively lower concentrations, whereas PFOA exhibited weaker structural effects. These findings are broadly consistent with prior evidence suggesting differential neurotoxic potential among PFAS subclasses [41,55]. Progressive neuronal damage over time further supports sustained dopaminergic vulnerability following exposure. Although the exposure concentrations exceed typical environmentally relevant levels, they are commonly used in toxicological studies to establish relative potency and characterize concentration-response relationships [41,55,61]. The derived BMC_10_ values provide a quantitative framework for cross-compound comparison of DA sensitivity.

Importantly, morphological degeneration was strongly associated with impairment of dopamine-dependent behaviors, including basal slowing response and area-restricted search. In some cases, behavioral deficits were detectable prior to extensive structural damage, suggesting that functional disruption may precede overt neuronal loss [62,63]. This pattern was particularly evident for PFOA, where basal slowing response was reduced beginning at 100 µM and area restricted search was reduced at 400 µM, both at concentrations lower than those at which clear phenotypic DA neuron degeneration was first observed. This finding is consistent with established models of dopaminergic dysfunction in which changes in synaptic activity or neurotransmission can occur before irreversible neuronal loss. The integration of imaging and behavioral endpoints therefore enhances sensitivity for detecting early neurotoxic effects.

For PFAS mixtures formulated with environmentally relevant component ratios, neurotoxic outcomes appeared to be more strongly influenced by the PFOS fraction than by total PFAS concentration (ΣPFAS). Mixtures containing lower proportions of PFOS showed attenuated neuronal and behavioral effects compared with PFOS-only exposures at equivalent total concentrations. These results support a composition-dependent toxicity profile in which a higher-potency constituent may exert a disproportionate influence on dopaminergic outcomes. Although clear synergistic interactions were not evident under the conditions tested, the findings highlight the importance of considering mixture composition, rather than total PFAS burden alone, in neurotoxicity evaluation [35,64].

Overall, this study establishes an efficient in vivo framework for assessing PFAS-induced DA neurotoxicity and demonstrates differential potency among individual PFAS and mixtures formulated with environmentally relevant component ratios. Although the current HTHC imaging platform was applied here to assess DA neuron integrity in the BZ555 strain, it could also be extended to other *C. elegans* models relevant to neurotoxicity and neurodegeneration. For example, strains such as NL5901 and UA44 could provide complementary insight into PFAS-induced neurotoxicity by allowing assessment of additional pathological features, including α-synuclein aggregation and α-synuclein-associated dopaminergic injury [32,65,66]. Applying this platform across multiple transgenic strains may help clarify whether PFAS primarily affects neuronal structure, dopaminergic function, proteostasis, or related pathways. This broader application would expand the utility of the platform and support more comprehensive mechanistic studies of environmental pollutant-induced neurotoxicity.

## 5. Conclusions

This study establishes a high-throughput, high-content (HTHC) in vivo platform for quantitative evaluation of dopaminergic neurotoxicity in *C. elegans*. Differential potency was observed among the individual PFAS tested, with PFOS exhibiting the strongest effects. In mixtures formulated with environmentally relevant component ratios, neurotoxicity was more strongly influenced by PFOS composition than by total PFAS concentration, supporting a composition-dependent toxicity profile. By integrating automated worm dispensing, simultaneous microfluidic immobilization, and Cytation 5 imaging, this workflow provides a rapid, standardized, and practical approach for dopaminergic neuronal assessment and may support broader application in environmental neurotoxicity screening.

## Figures and Tables

**Table 1:** Composition of PFAS mixtures and exposure concentrations.

Mixture ID	Components	Normalized Ratio *	ΣPFAS (µM)	PFOS (µM)	PFHxS (µM)	PFHxA (µM)	PFOA (µM)
M1	PFOS+	0.88:0.12	100	88.0	-	-	12.0
	PFOA		200	176.0	-	-	24.0
M2	PFOS+	0.60:0.30:0.10	100	60.0	30.0	-	10.0
	PFHxS+						
	PFOA		200	120.0	60.0	-	20.0
M3	PFOS+	0.56:0.28:0.09:0.07	100	56.3	27.6	9.2	6.9
	PFHxS+						
	PFHxA+						
	PFOA		200	112.6	55.2	18.4	13.8

* Ratios were normalized from the median environmental proportions (PFOS:PFHxS:PFHxA:PFOA = 0.49:0.24:0.08:0.06).

**Table 2:** The scoring criteria for the 6 head-region DA neurons in *C. elegans*.

Score	Criteria
0	6 perfectly healthy intact neurons *
1	5 intact neurons OR 6 intact neurons + morphological damage (kinks, blebs)
2	1–4 intact neurons
3	0 intact neurons

* Intact neuron refers to a visible pair of a soma and its corresponding axon.

**Table 3:** The Benchmark Concentration 10% PFAS on neuronal damage on day 10.

PFAS	PROAST Best Model	BMC (BMCL) [µM]	Akaike Information Criterion (AIC)
PFOS	Exponential—5	4.73 (0.97)	186.56
PFOA	Exponential—3	11.23 (0.27)	738.12
PFHxS	Exponential—5	33.01 (0.08)	421.14
PFHxA	Hill—5	113.3 (64.9)	461.52

## Data Availability

The original contributions presented in this study are included in the article. Further inquiries can be directed to the corresponding author.
